# Peripersonal space and interpersonal distance as windows onto multisensory body representation in adolescents with overgrowth syndromes

**DOI:** 10.3389/fnhum.2026.1951893

**Published:** 2026-09-08

**Authors:** Niccolò Butti, Rosario Montirosso, Alice Cartaud, Viola Oldrati, Yann Coello, Cosimo Urgesi

**Affiliations:** 1 Scientific Institute, IRCCS E. Medea, 0-3 Centre for the at-risk infant, Bosisio Parini, Lecco, Italy; 2 CNRS, UMR 9193 - SCALab - Sciences Cognitives et Sciences Affectives, University of Lille, Villeneuve d'Ascq, France; 3 CNRS, IESEG School of Management, UMR 9221 - LEM - Lille Économie Management, University of Lille, Lille, France; 4 Scientific Institute, IRCCS E. Medea, Laboratory of Neuroplasticity and Neuromodulation, Bosisio Parini, Lecco, Italy; 5 Scientific Institute, IRCCS E. Medea, Pasian di Prato, Udine, Italy; 6 Department of Human and Social Sciences, Universitas Mercatorum, Roma, Italy

**Keywords:** peripersonal space, interpersonal distance, overgrowth syndromes, autistic traits, body mass index, social challenges, social perception, adolescence

## Abstract

**Introduction:**

Peripersonal space (PPS) and interpersonal distance (IPD) reflect related but dissociable representations of the space around the body, both relevant to multisensory and social development. However, whether physical overgrowth is associated with an altered regulation of body-related space remains unknown, despite growing evidence of psychosocial difficulties in individuals with overgrowth syndromes.

**Methods:**

This study aimed to characterize PPS and IPD in adolescents with overgrowth syndromes, specifically Beckwith-Wiedemann spectrum, Sotos syndrome, and Malan syndrome, and to test whether their expansion is associated with syndrome type, cognitive functioning, body size, and social-perceptual ability. Thirty-three adolescents with overgrowth syndromes and 33 age-matched controls completed separate comfort-distance and reachability judgments for social and non-social stimuli using a computerized stop-distance paradigm.

**Results:**

Adolescents with overgrowth syndromes showed overall larger PPS and IPD than controls, independent of autistic traits (*p* < 0.001, *η*^2^*_p_* = 0.25). Within the clinical group, the relative expansion of IPD over PPS was larger in adolescents with Beckwith-Wiedemann spectrum (*t*_31_ = −2.57, *p* = 0.015) and in those with average intellectual functioning (*t*_31_ = −2.67, *p* = 0.012), pointing to a buffer space linked to feelings of safety. A larger buffer space was further predicted by better affect-recognition skills (*β* = 0.48, *p* = 0.010), independent of age and autism-like social challenges. The body mass index was selectively associated with PPS (*r* = 0.47, *p* = 0.006).

**Discussion:**

These findings indicate that body-related spatial representations are enlarged in overgrowth syndromes, and that this expansion is shaped by cognitive functioning, body size, and social-perceptual ability rather than by autistic traits, contributing novel evidence to the study of typical and atypical multisensory development and its association with social functioning. This may also inform interventions targeting social participation in adolescents with overgrowth syndromes.

## 1 Introduction

The brain creates multisensory representations of the space with reference to the body and its parts to perceive and interact with external stimuli in the environment. Peripersonal space (PPS) represents a particularly functional sector of space around the body, as it is where all physical interactions between the individual and the environment take place (Serino, 2019). PPS has its neurobiological foundation in the discovery of the so-called bimodal neurons, responding similarly to both somatosensory and visual stimuli only when they are presented in space regions near the body (Rizzolatti et al., 1981a; Rizzolatti et al., 1981b; Fogassi et al., 1996). These multisensory neurons link the sight of objects to expected tactile events, and are part of a wider fronto-parietal network, including the premotor cortex and the posterior parietal cortex (di Pellegrino and Làdavas, 2015; Geers and Coello, 2023). Due to these features, PPS is also referred to as the action space, a multisensory-motor interface that prepares the body for voluntary object-directed movements or for defensive actions in case of threat (Cartaud et al., 2018; Finisguerra et al., 2015). Although there are multiple body-part centered representations, there is consistent evidence of a global PPS surrounding the whole body (Serino et al., 2015). This global PPS partially overlaps with the body schema (Cardinali et al., 2009; Medina and Coslett, 2010), which directly encodes body size and position to plan and execute goal-directed movements (Canzoneri et al., 2013; D’Angelo et al., 2018), supporting a general representation of the self as distinct from the environment and from others.

A solid body of evidence has demonstrated that this multisensory representation of space is modulated by stimulus features, internal traits, tool use, as well as by emotional and social contexts (Serino, 2019; Ruggiero et al., 2021; Cartaud et al., 2022; Ferroni et al., 2025; Quesque et al., 2017). This flexibility is particularly important during social interactions, as regulating physical distance from other is essential to avoid discomfort and support effective communication (Perry et al., 2013). Building on seminal studies in proxemics (Hall, 1995; Hall et al., 1968; Sundstrom and Altman, 1976), the space individuals maintain between themselves and others during social interactions is often referred to as interpersonal distance (IPD; Patané et al., 2017; Candini et al., 2019). Although theoretical and empirical disputes continue about whether and to what extent PPS and IPD overlap (Serino, 2019; de Vignemont and Iannetti, 2015), prior research has examined these two spatial representations in the same participants. Starting from Iachini et al. (2014), a stream of research has adopted behavioral paradigms to test reachability and comfort distances, considered as proxies of PPS and IPD respectively, with respect to social and non-social stimuli (Cartaud et al., 2018; Ruggiero et al., 2021; Quesque et al., 2017; Iachini et al., 2016; Nandrino et al., 2017; Cartaud et al., 2020; Cartaud et al., 2024). It is to note, however, that reachability distance differs from PPS as measured in visuo-tactile paradigm (Zanini et al., 2021). These studies provide evidence that IPD is usually larger than PPS, as it contains the action space plus a ‘buffer space’ that contributes to feelings of comfort and safety (Coello and Cartaud, 2021). Although both spatial representations are influenced by social perception of others (Iachini et al., 2015; Pellencin et al., 2018), IPD appears to be more strictly related to social functioning (Perry et al., 2013; Candini et al., 2020), while PPS is primarily underpinned by sensorimotor representations and body size (Bassolino et al., 2012; Sugovic et al., 2016).

Altered regulation of PPS and IPD has been documented across neurodevelopmental disorders characterized by abnormal social behavior (Lough et al., 2015). Individuals with ASD have been found to exhibit atypical distance regulation, either maintaining excessive distance or intruding into others’ personal space (Candini et al., 2020; Gessaroli et al., 2013; Fusaro et al., 2023; Farkas et al., 2023). Notably, research in ASD has revealed a dissociation between action and social spaces, with IPD regulation being more specifically affected and correlated with the level of social impairment (Candini et al., 2019; Candini et al., 2017; Mul C lène et al., 2019). Biases in in the estimation of IPD were also found to be associated with socially inappropriate distance preferences in people with autism (Givon-Benjio et al., 2024). Furthermore, a failure of social space regulation has been described in Williams syndrome (Lough et al., 2015; Lough et al., 2016), a genetic condition presenting with hyper-sociable behavior and specific neuropsychological impairments (Frigerio et al., 2006; Miezah et al., 2020). In such disorders, abnormal IPD may result from poor processing of social cues, contributing to socio-communicative and behavioral problems (Candini et al., 2020). Overall, previous literature highlights a link between body-related spatial representations, processing of social cues, and challenges in social behavior (Bogdanova et al., 2021; Basile et al., 2024).

Overgrowth syndromes are a heterogeneous group of rare genetic disorders characterized by excessive growth of the whole body or of specific body regions (Brioude et al., 2019). Beckwith-Wiedemann spectrum (BWSp) is the most common overgrowth disorder, with an estimated prevalence of 1:10,500 newborns (Wang et al., 2020). It presents with a wide range of primary and secondary features, including macroglossia and hemihyperplasia (i.e., lateralized overgrowth), whereas cognitive impairments are not usually observed (Brioude et al., 2018). Psychosocial challenges have increasingly been documented in individuals with BWSp, particularly in relation to peer interactions, anxiety and social withdrawal (Butti et al., 2022; Butti et al., 2025c; D’Onofrio et al., 2024; Drust et al., 2023). Sotos syndrome is a rare autosomal dominant disorder, with an estimated prevalence of 1:14,000 newborns (Atterton et al., 2025). Its core features include physical overgrowth and cognitive impairments, although the severity of the phenotype can vary widely (Lane and Freeth, 2019). In Sotos syndrome, difficulties in social interactions and comorbid ASD are commonly described (Lane et al., 2019; Lane et al., 2017; Viswanathan et al., 2025), with a prevalence of mild to moderate autistic symptoms (Riccioni et al., 2024), despite social perception skills can be relatively preserved (Butti et al., 2025b). Malan syndrome is an ultra-rare disorder, occurring in fewer than 1 in 1,000,000 individuals (Huynh et al., 2024). Malan syndrome shares cardinal clinical features with Sotos syndrome, as suggested by its previous definition as Sotos-2 or Sotos-like syndrome, but typically presents with moderate-to-severe intellectual disability (Macchiaiolo et al., 2022; Butti et al., 2025a). In Malan syndrome, behavioral challenges often include heightened anxiety, reduced social attention, and autism-like behaviors, though clinical signs of ASD appear to be limited (Alfieri et al., 2023). Difficulties of visual-motor integration and in sensory processing are also common features in both Sotos and Malan syndromes (Mulder et al., 2020; Smith et al., 2023). Because the body schema that underlies PPS directly encodes body size and proportions (Cardinali et al., 2009; Holmes and Spence, 2004), such alterations in growth and motor integration provide a plausible mechanistic link between the overgrowth phenotype and an atypical calibration of body-centered spatial representations. Furthermore, as BWSp, Sotos and Malan syndromes present structural body abnormalities from birth, but differ markedly in growth patterns and neurodevelopmental profiles (Brioude et al., 2019), these conditions can be seen as distinct yet complementary models to explore the interplay between atypical bodily features, neuropsychological functioning, and social behavior and how these influence multisensory representations of the space. Despite this body of evidence, whether and how overgrowth conditions are associated with alterations in body-centered spatial representations remains largely unexplored, and no previous study has examined PPS and IPD within the same paradigm in these populations.

With this aim, in the present study adolescents with overgrowth syndromes and healthy peers were administered a computer-based stop-distance paradigm to assess PPS and IPD for social and non-social stimuli (Ruggiero et al., 2021; Iachini et al., 2014). Adolescence represents a critical developmental stage for body-related representations, as pubertal changes and increasing salience of bodily appearance shape peer interactions and social behavior (De Witte et al., 2016; Raoul and Grosbras, 2023). Furthermore, recent findings indicate that IPD decreases and stabilizes during the transition from childhood to adolescence, likely reflecting developmental changes in social cognition (Mirlisenna et al., 2024). This age range thus represents a sensitive window for examining body-centered spatial representations in overgrowth syndromes.

As a first level of analysis, the comparison with typically developing peers was intended to establish whether multisensory body-centered representations in adolescents with overgrowth syndromes differ from those observed in typical development. Subsequent analyses focused on the clinical sample to examine factors associated with variability in IPD and PPS. Because BWSp differs substantially from Sotos and Malan syndromes in clinical features (Brioude et al., 2019; Atterton et al., 2025), we examined potential syndrome-specific differences in comfort- and reachability-distance judgments. We also tested whether adolescents without cognitive impairments differed from their peers with cognitive impairments to examine potential effects of cognitive functioning on task performance (Palix et al., 2020). At this level of analysis, we also considered body mass index (BMI) to account for possible effects of body size. Lastly, as a third, exploratory level of analysis, we examined whether age, social perception skills, and autism-like social challenges were associated with a differential expansion of comfort relative to reachability distance. Building on the multisensory-motor account of PPS and on evidence of different neurodevelopmental profiles across overgrowth syndromes, we hypothesized that (i) adolescents with overgrowth syndromes would show an overall expansion of both PPS and IPD compared with typically developing peers; (ii) this expansion would vary across syndromic groups and according to cognitive functioning, reflecting distinct contributions of bodily, motor, and cognitive factors to space regulation; and (iii) body size (BMI) would be more closely related to PPS, whereas social-perceptual skills would be more closely related to IPD.

## 2 Methods

This study adopted a cross-sectional, quantitative, case–control design, comparing adolescents with genetically confirmed overgrowth syndromes to typically developing peers on a computerized stop-distance paradigm. This behavioral approach was chosen because it allows comfort- and reachability-distance judgments, as validated proxies for IPD and PPS, respectively, to be obtained within the same standardized procedure and directly compared across social and non-social stimuli (Iachini et al., 2016; Cartaud et al., 2024). The following sections describe participants, procedures, and the statistical approach used to test the study hypotheses.

### 2.1 Participants and procedures

Participants with overgrowth syndromes were recruited as part of a broader project investigating their cognitive and social functioning. With the support of the Italian family associations for BWSp (AIBWS), Sotos syndrome, and Malan syndrome (ASSI Gulliver), interested families were contacted to schedule their visit to the Scientific Institute IRCCS *E. Medea*, where assessments were conducted either during a short hospitalization (approximately 1 week) or over two consecutive days for day-hospital evaluations, depending on the families’ needs and availability. For the purposes of the present study, we considered clinical data collected during these assessments, including autistic traits and autism-like social challenges assessed using the Autism Quotient (AQ) questionnaire, performance at social perception subtests assessing theory of mind and affect recognition skills (see the sections below for details), and BMI. Height and weight were measured directly during clinical assessments for participants admitted for short hospitalizations, or obtained from parent reports and verified against the most recent clinical records for those attending day-hospital evaluations. BMI was then calculated according to the standard formula.

Thirty-three adolescents aged 11–18 years with overgrowth syndromes participated in the study (16 females, mean age = 14.3 years, SD = 2.4). Among them, 11 had a diagnosis of BWSp, 19 had Sotos syndrome and 3 had Malan syndrome. Inclusion criteria were: (i) a confirmed genetic diagnosis of overgrowth syndrome, (ii) age ranging from 11 to 18 years, and (iii) absence of severe motor and visual impairments that could interfere with task execution. A review of clinical records provided information on each participant’s level of intellectual functioning; however, the use of different instruments (e.g., Leiter scales, Raven’s Progressive Matrices, Wechsler scales) for testing intelligence quotient (IQ) precluded its inclusion in the analyses. Specifically, among participants with Sotos syndrome, five had an intellectual disability (IQ < 70) and eight exhibited borderline intellectual functioning (IQ 70–84); the remaining six had average intellectual abilities (IQ ≥ 85). As expected, all adolescents with Malan syndrome presented with intellectual disability, whereas only one individual with BWSp showed borderline intellectual functioning. Moreover, four adolescents with Sotos syndrome and one with BWSp had a documented comorbid diagnosis of ASD.

Adolescents with typical development were recruited as a control sample. Inclusion criteria were: (i) age 11–18 years, (ii) having normal or corrected-to-normal vision (with glasses or contact lenses), (iii) having no history of or any form of neurological and psychiatric disorders, and of medical or developmental conditions affecting growth or weight. Thirty-three participants were included in the control group. All participants, in both the clinical and control groups, were recruited in Italy, and therefore share a broadly comparable cultural background with respect to proxemics norms. According to their age, participants (>15 years) or their parents compiled the Autism Quotient (AQ; see the section below for details) questionnaire concerning the presence of autistic traits. Demographic and clinical information for the participants with overgrowth syndromes and control adolescents is presented in Table 1.

**Table 1:** Demographic and clinical characteristics of adolescents with overgrowth syndromes and control participants.

Variable	Overgrowth syndromes			Controls
	BWSp	Sotos syndrome	Malan syndrome	
Sex (male: female)	3:8	12:7	2:1	14:19
Age (years, mean, SD)	13.4 (2.3)	14.5 (2.3)	17.1 (2.4)	13.4 (2)
Genetic diagnosis (N)	Gain of methylation at the IC1 (2); Loss of methylation at the IC2 (8); UPD(11)pat (2)	Intragenic mutation of NSD1 (12); Microdeletion of NSD1 (5)	Intragenic variants of NFIX (2); Microdeletion 19p13.2—687–793 Kb (1)	
BMI (mean, SD)	18.5 (2.4)	19.7 (3.4)	18.5 (4.1)	
Cognitive impairments (N)	1	13	3	0
Autism Spectrum Disorder (N)	1	4	0	0
AQ Total (mean, SD)	58 (17)	73 (22)	80 (6)	57 (13)
AQ Social challenges (mean, SD)	9 (3)	12 (6)	16 (4)	8 (4)

BWSp = Beckwith-Wiedemann spectrum; IC1 = imprinting centre 1; IC2 = imprinting centre 2; UPD(11)pat = uniparental paternal disomy of the 11p15.5 chromosomal region; BMI = body mass index; AQ = autism quotient.

Age and sex assigned at birth did not differ significantly between the control and overgrowth-syndromes groups (*t*_64_ = 1.70, *p* = 0.095; *X*^2^ = 0.55, *p* = 0.459). As expected by the comorbidities with ASD, participants with overgrowth syndromes showed higher levels of autistic traits than healthy peers (*t*_64_ = 2.62, *p* = 0.011), particularly among those with Sotos and Malan syndromes, despite considerable variability was observed. The syndromic groups did not differ significantly in age, BMI, or AQ scores (all *p* > 0.057). Although BMI was not available for the control group, the inclusion criteria excluded medical or developmental conditions affecting growth or weight, suggesting that BMI values were likely within the normative range.

Participants were administered the stop-distance paradigm, developed on the basis of previous research (Cartaud et al., 2020; Iachini et al., 2014), in a quiet room. The stimuli were presented by means of a 15.4-inch LCD monitor (resolution 1024 × 768 pixels) kept at eye distance of approximately 50 cm. The monitor was connected to a laptop PC running E-Prime 3® software, which controlled for task administration and response collection. All procedures required about 30 min to be completed; they were in accordance with the Declaration of Helsinki and its later amendments, and were approved by the Ethical Committee of the Scientific Institute, IRCCS *E. Medea* (Prot. 18/21 CE). In both groups, participants and their parents were informed about aims and methods of the study before starting any procedure, and parents were asked to sign an informed consent form.

### 2.2 Virtual stop-distance paradigm

Two tasks were administered to assess comfort- and reachability distance, which are considered as proxies for IPD and PPS, respectively. The two tasks were presented as separate blocks of a single experiment, in which participants were shown the same videos but received different instructions. The videos were shown in a first-person perspective and displayed an approaching stimulus in an empty room. For the comfort-distance block, participants were asked to press the space bar when the virtual stimulus was at a comfortable distance to interact with. For reachability judgments, participants were instructed to press the space bar when they perceived the stimulus to be located at a virtual distance that could be effortlessly reached with their hand (e.g., touch the shoulder).

The approaching stimuli were a red or a blue cylinder, representing non-social objects, a male or a female human character, considered as social stimuli, and a light or a dark robot, hybrid figures which retained the kinematic features of human movement. Each stimulus type was presented an equal number of times. Stimuli appeared at the end of the room, corresponding to a virtual distance of 404 cm from the participants’ perspective, and then approached them with a constant velocity of 40 cm/s. The cylinder movement was created by means of a presentation of 47 pictures with an 8-cm step between each picture. Walking movements of the human and robot stimuli simulated the natural swing of biological motion. The approach of all stimuli, including the human and robot figures, followed a linear trajectory at constant velocity rather than a naturalistic, decelerating gait cycle; the looming expansion of the stimulus on the screen was therefore driven solely by its increasing proximity, without additional cues such as gait-related deceleration.

**Figure 1:**
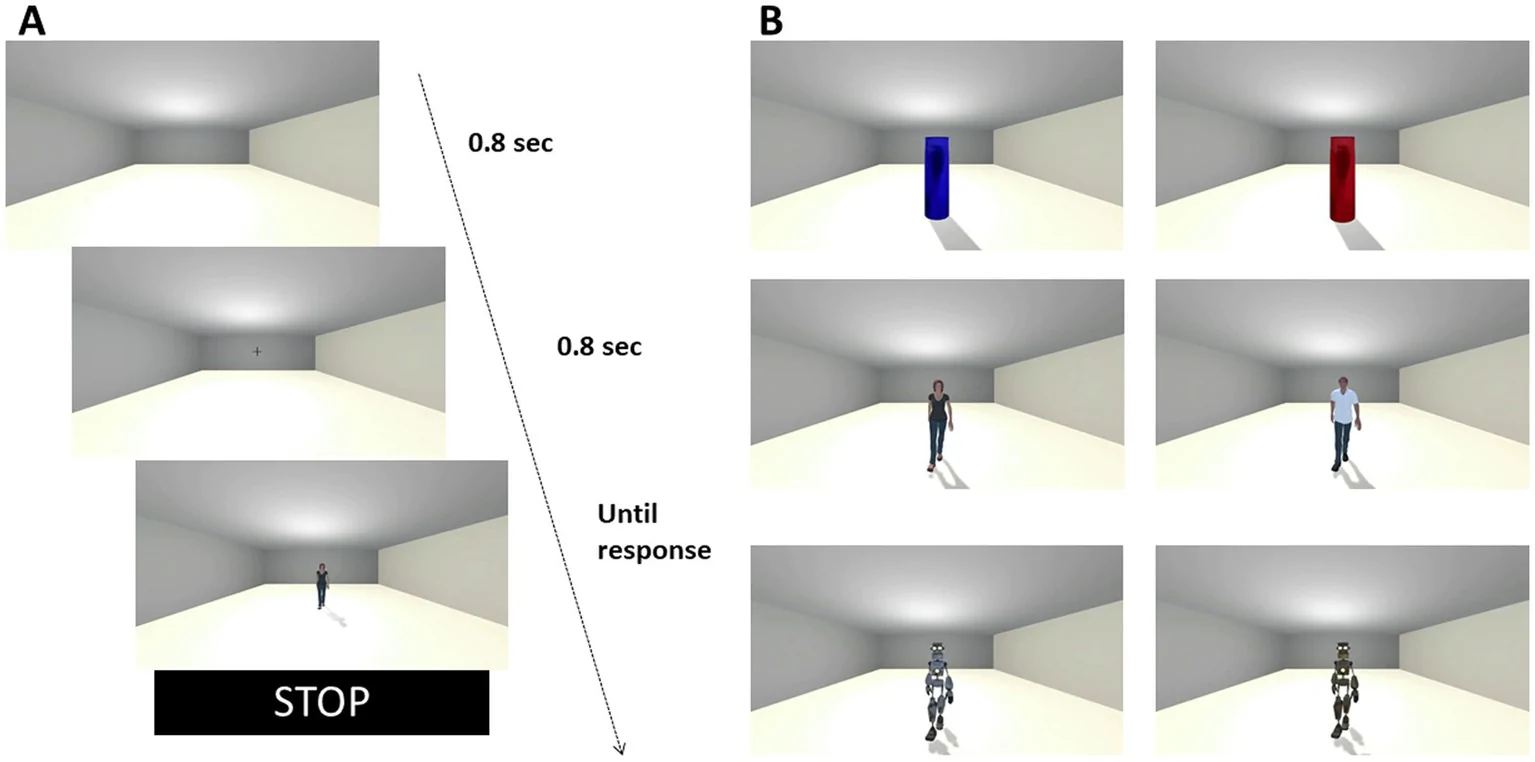
Timeline and stimuli used in the stop-distance paradigm tasks. **(A)** Timeline of the trials; **(B)** Stimuli for each category (from top to bottom: cylinder, human character, robot).

Each trial started with a frame displaying the empty room for 0.8 s, followed by a centered fixation cross. The stimulus then appeared and started to approach the participant until he or she pressed the space bar, which triggered the next trial (Figure 1). If the participant did not press the bar, the stimulus disappeared when it was 28 cm from the participant’s perspective and the response was not recorded.

Each stimulus was randomly presented 10 times per block (comfort-distance and reachability judgment) leading to a total of 60 trials in each block. Order of blocks was counterbalanced across the participants of each group. Each block lasted about 10 min, with a short break allowed in between.

The task script and stimuli are freely available from OSF at the following link: https://osf.io/56kuc/overview?view_only=921cc633d286498dba42bc1229966337.

### 2.3 Assessment of autistic traits

In both clinical and control groups, the Italian version of the AQ was administered to assess traits associated with ASD (Ruta et al., 2012; Baron-Cohen et al., 2006; Auyeung et al., 2008). The AQ is a 50-item self- or parent-report questionnaire that evaluates five areas of autism-like behaviors, with answers provided on a 0–3 Likert scale. The 10 items of each scale are summed up to obtain a score of autistic traits in that dimension (score range 0–30). A total AQ is also calculated by summing all scale scores (score range 0–150). Here, as the main interest was on social functioning, only the Social Skills scale and the total AQ were considered, with higher scores in these variables representing greater challenges in social interaction and higher general autistic traits, respectively. It is important to note that the AQ was developed for research purposes and, as such, should not be regarded as a diagnostic tool for ASD (Baron-Cohen et al., 2006).

### 2.4 Social perception

The Affect Recognition and Theory of Mind subtests from the NEPSY-II battery (Korkman et al., 2007; Urgesi et al., 2011) were administered exclusively to adolescents with overgrowth syndromes, as part of a broader neuropsychological assessment. In the Affect Recognition subtest, participants were required to discriminate and match facial expressions of emotion. The Theory of Mind subtest involved short stories or illustrated scenarios designed to assess participants’ abilities to adopt another individual’s perspective, infer mental states, and determine the appropriate emotional response within specific social contexts. Raw scores from both subtests were converted into scaled scores (mean = 10, SD = 3, range = 1–19) based on normative standardization tables.

### 2.5 Data handling and statistical analysis

For each trial, comfort and reachability distances (in meters, m) were calculated using the recorded response time, starting distance, starting time, and stimulus velocity, according to the following formula:

$$\text{Distance}=\text{starting distance}-((\begin{array}{l} \text{response time}\\ -\text{starting time} \end{array})\times \text{velocity})$$

To examine whether body-related spatial representations differed between adolescents with overgrowth syndromes and typically developing peers, task performance was first compared across groups. Comfort and reachability distances were inserted into a 2 × 2 × 3 ANCOVA with group (overgrowth syndromes vs. control) as a between-subject factor, task (IPD vs. PPS) and stimulus (cylinder, human, robot) as within-subject variables. Total AQ score was included as a covariate to account for the observed between-group differences in autistic traits.

Subsequently, we investigated whether variability in PPS and IPD within the overgrowth group was associated with syndrome-specific characteristics or cognitive level, while controlling for BMI as a proxy of body size. First, participants with Sotos syndrome and with Malan syndrome were combined into a single group, and compared with participants with BWSp. Combining Sotos and Malan syndromes into a single syndromic group was a pragmatic choice, dictated primarily by the very small number of participants with Malan syndrome (N = 3), although these conditions share many primary features and are both classified as overgrowth-intellectual disability disorders (Atterton et al., 2025). The two syndromic groups were entered as a categorical factor in a 2 × 2 × 3 ANCOVA, with task and stimulus as within-subject variables and BMI as a covariate. Second, adolescents with overgrowth syndromes were split into two subgroups, one with an average or above-average intellectual level, and one with cognitive impairments, including individuals with intellectual disability and borderline intellectual functioning. The subgroup with average intellectual level was composed by six adolescents with Sotos syndrome and 10 with BWSp (5 males; age mean = 13.8, SD = 2.3), while the subgroup presenting cognitive impairment consisted of three participants with Malan syndrome, one with BWSp, and 13 with Sotos syndrome (12 males; age mean = 14.9, SD = 2.5). While age, BMI, and total AQ did not differ between these subgroups (all *p* > 0.149), a significant difference emerged in sex distribution (*X*^2^ = 5.11, *p* = 0.024). Therefore, we preliminary examined the potential impact of sex assigned at birth by inserting it as a categorical factor in a 2 × 2 × 3 ANOVA, with task and stimulus as within-subject variables. After ruling out this potential confounder (see Supplementary material for results), a subsequent ANCOVA was conducted, with task and stimulus as within-subject variables, cognitive level as a between-subject factor, and BMI as a covariate. Duncan’s post-hoc tests were used to explore significant interactions with correction for multiple comparisons. The direction of significant covariate effects was tested through Pearson’s *r* correlations with averaged distances in the two tasks.

A Buffer Space (BS) index was calculated according to the following formula:

$$\begin{array}{c} \mathrm{BS}=\text{comfort distance from human characters}\\ -\text{reachability distance from human characters} \end{array}$$

This index operationalized the homeostatic model of the interrelation between peripersonal action space and interpersonal social space proposed by Coello and Cartaud (2021), according to which IPD reflects the boundary of PPS plus an adjustable safety margin that offers a sense of security and comfort during social interaction (Coello and Cartaud, 2021). Higher values indicated an expansion of comfort with respect to reachability distance. Group differences in BS were examined using independent-samples *t*-tests. Specifically, BS values were compared (i) between adolescents with overgrowth syndromes and typically developing peers, (ii) between BWSp and Sotos/Malan syndromes, and (iii) between individuals with average intellectual level and those with cognitive impairments. To control for multiple comparisons across these three tests, Bonferroni-adjusted *p* values were applied (*α* = 0.05/3).

Finally, we examined associations between the BS index, age, and measures of social functioning using a multiple regression model. Prior to analysis, data were inspected for potential violations of model assumptions. Plots of standardized residuals versus predicted values showed no clear pattern, indicating linearity and homoscedasticity of residuals. All Cook’s distance values were below 0.5, suggesting the absence of influential outliers. Based on the observed correlations (see Supplementary Table S1), age, the AQ Social Skills scale, and scaled scores at the Affect Recognition subtest were retained as predictors in the model, with BS values entered as the dependent variable.

To verify the robustness of the findings and that grouping Malan and Sotos syndromes did not obscure syndrome-specific effects, all analyses were also repeated after excluding the Malan subgroup (see Supplementary material).

All analyses were performed with Statistica 8.0 (Statsoft, Tulsa, OK), with the significance threshold set at *p* < 0.05 for all effects. Data are reported as mean ± SEM; regression coefficients are reported with 95% confidence intervals, computed from standard errors and the appropriate *t*-distribution. Effect sizes were reported as partial eta squared (*η*^2^*_p_*) for ANOVA designs, adopting conventional cut-off of 0.01, 0.06, and 0.14, for small, medium, and large effect sizes, respectively (Lakens, 2013).

For the main comparison between clinical and control groups, an a-priori power analysis was conducted using the G*Power 3 software (Faul et al., 2007) with the “as in SPPS” option, setting the alpha-level at 0.05 and desired power at 95%. According to previous research adopting the same behavioral tasks (Iachini et al., 2014) and investigating PPS and IPD in typically developing and autistic children (Candini et al., 2019), we expected a medium-to-large effect size [*η*^2^*_p_* = 0.12; *f*(U) = 0.369]. For a 2-group ANOVA with 3 × 2 repeated measures (numerator df = 2), a total sample of at least 62 participants (31 per group) was deemed sufficient to detect such effects.

## 3 Results

### 3.1 Comfort and reachability distances in adolescents with overgrowth syndromes compared to control peers

The ANCOVA revealed a significant main effect of group (*F*_1,63_ = 20.98, *p* < 0.001, *η*^2^*_p_* = 0.25), indicating that the group with overgrowth syndromes preferred larger distances than the control participants (2.07 ± 0.08 vs. 1.50 ± 0.08 m). The main effects of task (*F*_1,63_ = 4.70, *p* = 0.034, *η*^2^*_p_* = 0.07) and stimulus (*F*_2,124_ = 31.48, *p* < 0.001, *η*^2^*_p_* = 0.34) were also significant. These findings confirmed that reachability distances were shorter than comfort distances for all stimuli (1.50 ± 0.06 vs. 2.07 ± 0.08 m) and that, across the two tasks, larger distances were kept from cylinders (1.90 ± 0.06 m) compared with robots (1.78 ± 0.06 m) and human characters (1.67 ± 0.06 m). Distances for robots were larger than those for human characters (all *p* < 0.001). The stimulus x group interaction was also significant (*F*_2,126_ = 3.91, *p* = 0.022, *η*^2^*_p_* = 0.06), showing that in the control group there was no difference between robot and other stimuli (*p* > 0.062). All stimuli comparisons were significant in the overgrowth syndrome group (all *p* < 0.001). Importantly, the preference (i.e., shorter distance) for human characters compared with the cylinders was detected in both groups (all *p* < 0.001). Neither the main effect of total AQ nor its interactions were significant (all *p* > 0.425), confirming that the between-group differences were not accounted by autistic traits.

### 3.2 Comfort and reachability distances across syndromic groups

The ANCOVA revealed a significant main effect of task (*F*_1,30_ = 9.43, *p* = 0.005, *η*^2^*_p_* = 0.24), with overall larger comfort distances than reachability distances. The main effect of group was not significant (*F*_1,30_ = 0.02, *p* = 0.895, *η*^2^*_p_* = 0.00), whereas the task × group was significant (*F*_1,30_ = 9.17, *p* = 0.005, *η*^2^*_p_* = 0.23). Duncan post-hoc tests confirmed larger IPD than PPS both in the BWSp group (2.44 ± 0.17 vs. 1.60 ± 0.13 m; *p* < 0.001) and in individuals with Sotos and Malan syndromes (2.26 ± 0.12 vs. 1.93 ± 0.09 m; *p* = 0.005). Although no between-group differences emerged (IPD: *p* = 0.329; PPS: *p* = 0.082), the significant interaction suggested a differential expansion of comfort relative to reachability distance in the two groups (see below Buffer space comparisons across groups). Neither the main effect of BMI nor its interactions reached significance (all *p* > 0.07).

### 3.3 Comfort and reachability distances in adolescents with average intellectual level compared to peers with cognitive impairments

**Figure 2:**
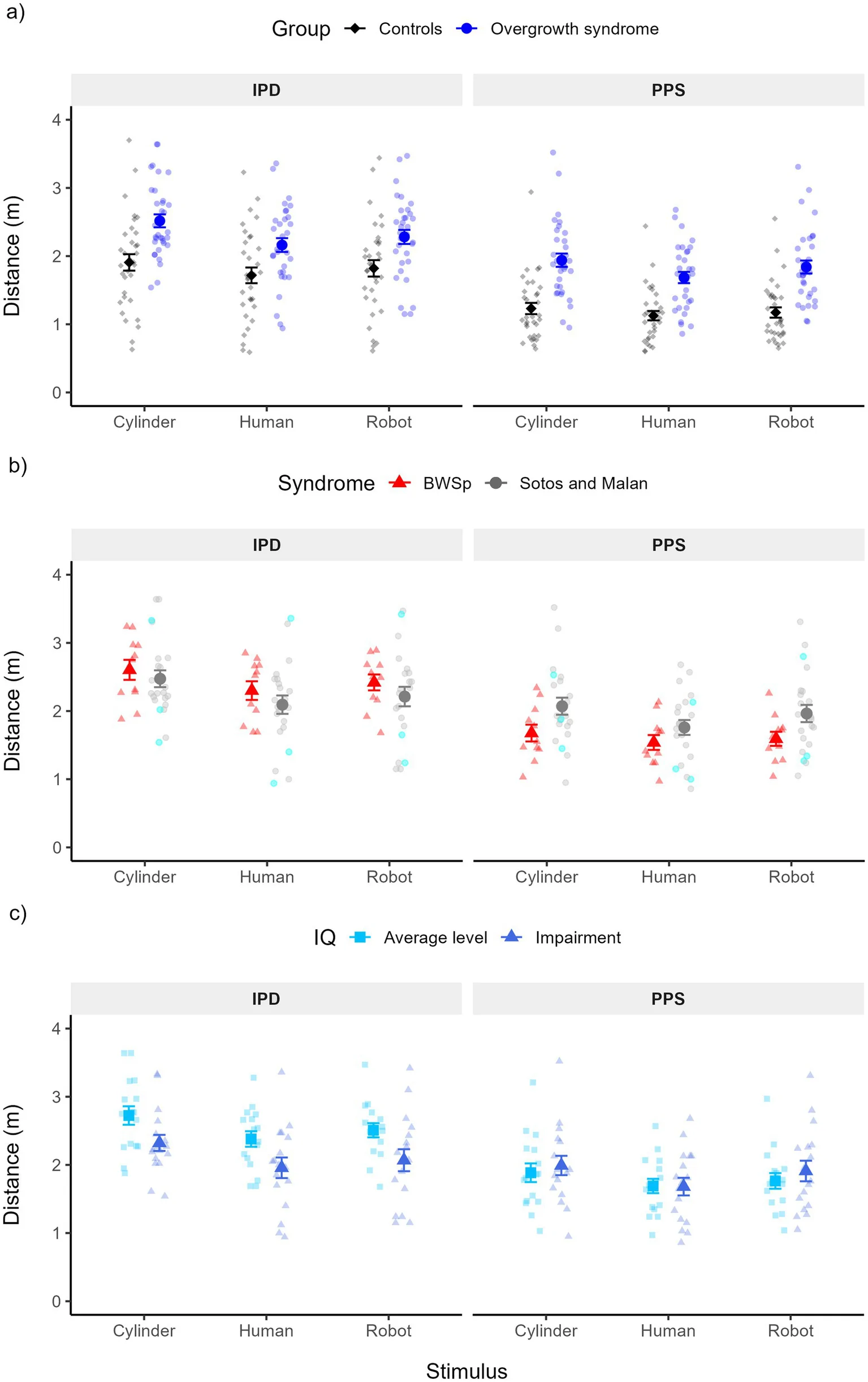
Interpersonal distance (IPD) and peripersonal space (PPS) in overgrowth syndromes and control participants **(a)** in Beckwith–Wiedemann Spectrum (BWSp) versus Sotos and Malan syndromes **(b)** and in adolescents with overgrowth syndromes with average intellectual level and with cognitive impairments **(c)**. Error bars represent SEM. In panel **(a)**, colored rhombi and dots indicate individual performances of control participants and adolescents with overgrowth syndromes, respectively. In panel **(b)**, colored triangles and dots indicate individual performance of participants with BWSp and with Sotos or Malan syndromes, respectively, with light blue dots identifying individual performances of participants with Malan syndrome. In panel **(c)**, colored squares and triangles indicate individual performances of participants with average intelligence quotient (IQ ≥ 85) and with cognitive impairments (IQ < 85), respectively.

When splitting the group with overgrowth syndromes according to intellectual level, the main effect of task (*F*_1,30_ = 7.63, *p* = 0.01, *η*^2^*_p_* = 0.20) was still significant, along with the task × group interaction (*F*_1,30_ = 9.92, *p* = 0.003, *η*^2^*_p_* = 0.25). Duncan’s post-hoc tests showed that adolescents with average intellectual level preferred larger comfort distances (2.54 ± 0.13 m) than those with cognitive impairments (2.12 ± 0.12 m; *p* = 0.016), whereas no group difference was found for reachability-distance judgments (1.78 ± 0.13 vs. 1.86 ± 0.12 m; *p* = 0.636). A significant effect of BMI also emerged (*F*_1,30_ = 5.09, *p* = 0.031, *η*^2^*_p_* = 0.15), indicating that body size influenced body-related spatial representations. Follow-up correlations revealed a significant positive association between BMI and reachability distance (*r* = 0.47, *p* = 0.006), but not with comfort distance (*r* = 0.14, *p* = 0.45). However, all its interactions with group were non-significant (all *p* > 0.114), confirming that variations in BMI did not account for the observed difference in IPD between individuals with average intellectual level and those with cognitive impairments (Figure 2).

### 3.4 Buffer space comparisons across groups

No significant difference in BS emerged between adolescents with overgrowth syndromes and typically developing peers (*t*_64_ = −0.88, *p* = 0.382). Within the clinical sample, BS differed significantly between syndromic groups. Specifically, participants with BWSp showed larger BS values than those with Sotos/Malan syndromes (*t*_31_ = −2.57, *p* = 0.015), an effect that remained significant after Bonferroni correction. BS values also differed by cognitive level. Adolescents with average intellectual functioning showed larger BS than those with cognitive impairments (*t*_31_ = −2.67, *p* = 0.012), which likewise remained significant after Bonferroni correction. This pattern is consistent with the ANCOVA results showing group differences primarily in IPD rather than PPS.

### 3.5 Multiple regression model on buffer space

The multiple regression model on BS was significant (Adjusted *R*^2^ = 0.18, *p* = 0.035). Among the predictors, only Affect Recognition significantly predicted BS (*β* = 0.48, *p* = 0.010, 95% CI [0.013, 0.092]), whereas age (*β* = −0.13, *p* = 0.455, 95% CI [−0.093, 0.043]) and AQ Social Skills (*β* = 0.19, *p* = 0.261, 95% CI [−0.013, 0.047]) were not significant. These results indicated that higher affect recognition ability was associated with a larger expansion of comfort distance relative to reachability distance, independently of age and autism-like social difficulties.

### 3.6 Supplementary analyses without Malan syndrome

When the subgroup with Malan syndromes was excluded, all analyses yielded results consistent with those obtained in the full sample. A trend toward greater PPS in Sotos syndrome compared with BWSp was observed (*p* = 0.051; please see Supplementary material for a detail description of the results).

## 4 Discussion

Adolescents with overgrowth syndromes showed larger PPS and IPD than typically-developing peers, suggesting an influence of physical overgrowth on body-related spatial representations. Consistent with prior studies using comparable paradigms (Geers and Coello, 2023; Iachini et al., 2014), both groups preferred shorter distances for human compared to the other stimuli. Although social challenges are reported across the three overgrowth syndromes, autistic traits did not account for the observed between-group differences. These findings are consistent with our first hypothesis, and suggest that the mechanisms underlying the general expansion of body-related spatial representations in overgrowth syndromes may differ from those implicated in ASD (Candini et al., 2020; Fusaro et al., 2023). One possible concern is that between-group differences might reflect the presence of cognitive impairments in a substantial proportion of adolescents with overgrowth syndromes, potentially affecting task performance. However, lower intellectual functioning is generally associated with longer reaction times (Palix et al., 2020), which would yield shorter, not longer, measured distances. Subsequent analyses within the clinical sample therefore aimed to clarify sources of variability in PPS and IPD beyond this general group difference.

When comparing the two syndromic groups (BWSp vs. Sotos and Malan syndromes), no direct between-group differences emerged in comfort- and reachability-distance judgments. However, the significant task × group interaction indicated a different modulation of IPD and PPS across syndromes. This pattern was clarified by the follow-up analysis on the BS index, which showed a larger relative expansion of comfort with respect to reachability distance in adolescents with BWSp. Thus, while absolute PPS and IPD values were broadly comparable across syndromic groups, their relative expansion differed, pointing to syndrome-specific modulation of social distancing rather than uniform shifts in spatial boundaries.

A convergent pattern emerged when the clinical group was subdivided based on intellectual functioning. Adolescents with average intellectual level exhibited larger IPD, but similar PPS, compared with those with cognitive impairments, further suggesting that the enlargement of PPS or IPD was not merely due to intellectual disability or spatial attention effect (Makin et al., 2012; Zanini et al., 2021). These findings also support the notion that PPS and IPD, although related, represent dissociable constructs, as previously demonstrated in both healthy and clinical populations (Patané et al., 2017; Candini et al., 2019; Coello and Cartaud, 2021). Consistently, the BS index was larger in individuals with average intellectual functioning, mirroring the pattern observed in adolescents with BWSp and suggesting a shared modulation of comfort distance across syndromic and cognitive-level analyses. Moreover, variations in BMI did not account for the differences in IPD across syndromic and cognitive-level subgroups. Taken together, these findings suggest that differences in the relative expansion of comfort versus reachability distance were not solely driven by syndrome-specific bodily features or by overall body size. Instead, they point to a potential role of cognitive functioning in shaping social spacing. This pattern is in line with our second hypothesis and suggests that, beyond gross anatomical differences, individual variation in cognitive resources may shape how the buffer space around the body is calibrated, possibly through fronto-parietal circuits implicated in the top-down regulation of body-related spatial representations (Basile et al., 2024).

When individuals with Malan syndrome, who presented a more severe phenotype, were excluded, a trend toward larger PPS in Sotos syndrome compared with BWSp was detected. Such an expansion of reachability distance may relate to greater arm span typically reported in Sotos syndrome (Atterton et al., 2025); however, as arm length was not measured in the present study, this interpretation remains speculative and should be addressed in future research. Importantly, in line with our hypothesis, BMI was positively associated with PPS but not with IPD, suggesting that body size may selectively relate to peripersonal action space representations. Similar adaptations in PPS have been observed in other contexts involving bodily changes, such as during pregnancy, when the sensorimotor representation of space adjusts to accommodate the enlarged body shape (Cardini et al., 2019).

The regression analysis further showed that stronger facial affect recognition skills, but not autism-like social challenges, were associated with a larger expansion of comfort distance. In individuals with ASD, atypical processing of social stimuli has been proposed to underlie the observed avoidance of physical proximity and social difficulties (Candini et al., 2020; Massaccesi et al., 2021). In contrast, adolescents with overgrowth syndromes and average intellectual level may even exhibit a heightened capacity to interpret others’ emotions (Butti et al., 2025c), yet still choose to maintain larger comfort distances.

In a more speculative perspective, the selective enlargement of comfort distance among adolescents with overgrowth syndromes and preserved intellectual functioning, particularly those with BWSp, may reflect higher-order socio-cognitive mechanisms (Pellencin et al., 2018; Sun et al., 2023). These individuals may be more aware of their atypical bodily features and of the challenges they experience in social contexts (Stor et al., 2022). This interpretation aligns with the homeostatic theory of social interactions (Coello and Cartaud, 2021), which posits that the optimal IPD corresponds to the boundaries of PPS plus an additional safety margin that dynamically adjusts according to the perceived valence or threat level of conspecifics. Within this framework, enlarged IPD may represent a protective response to social context. Supporting this view, a recent study reported larger IPD in deaf individuals than in hearing participants, suggesting that increased distance could reflect a response to perceived social exclusion (Arioli et al., 2025). Previous studies have also linked IPD regulation to fear of negative social evaluation (Finn et al., 2024; Alcaraz-Ibáñez et al., 2023), emotional expressions conveyed by facial cues (Vieira et al., 2017), and approach-avoidance responses related to moral judgments (Cartaud et al., 2020). In this vein, repeated experiences of being stared at or commented upon because of an atypical appearance could lead to a protective widening of interpersonal space. However, alternative, non-mutually-exclusive explanations for the present pattern should also be considered. For instance, motor coordination difficulties documented in Sotos and Malan syndromes could inflate reachability and comfort judgments independently of any higher-order socio-cognitive mechanisms. Distinguishing between these accounts will require future studies combining behavioral distance judgments with direct measures of multisensory integration and of appearance-related social anxiety.

Noteworthy, evidence on emotional-behavioral and social challenges in BWSp is mostly derived from parent-report measures collected in relatively small samples (Butti et al., 2022; D’Onofrio et al., 2024). Within this context, the present findings contribute to the literature by providing task-based evidence of increased social distancing in adolescents with BWSp, and in those with Sotos syndrome and preserved intellectual functioning.

The study has limitations that should be discussed. Consistent with the rarity of overgrowth syndromes, the clinical sample was relatively small and unevenly distributed across syndromic groups, which are characterized by distinct growth patterns and neurodevelopmental profiles. This imbalance may limit the generalizability of the findings. Nonetheless, the multi-layer analysis approach reported consistent findings, and supplementary analyses confirmed the robustness of the main results. Cognitive functioning was assessed clinically using different standardized instruments, which limited the precision of between-participant comparisons and prevented its inclusion in the analyses as a continuous covariate. A further limitation concerns the cultural homogeneity of the sample. Both the clinical and control groups were recruited in Italy, and proxemic norms are known to vary substantially across cultures (Sorokowska et al., 2017; Wei et al., 2024). Future cross-cultural replications would help establish whether the alterations described here are universal correlates of overgrowth syndromes or are modulated by cultural context. Concerning the stop-distance paradigm, the administration of the tasks through a computer monitor reduces the ecological validity of the results compared with lab-based and virtual-reality settings (Candini et al., 2019; Simões et al., 2020). However, the use of monitor-presentation modalities for the virtual-reality scenarios limited the potential sickness that is associated with head-mounted displays and may affect performance in participants with cognitive impairments. Although reaching-distance tasks are commonly used as proxies for estimating PPS boundaries (Coello and Cartaud, 2021), this study did not include a direct assessment of PPS through established paradigms, such as visual-tactile and audio-tactile integration tasks, in-person stop-distance procedures, or tool-use paradigms (Finisguerra et al., 2015; Canzoneri et al., 2013; Dijkerman and Farnè, 2015; Bassolino et al., 2015). Integrating multiple PPS measures in future research would make it possible to disentangle defensive and goal-directed action components (de Vignemont and Iannetti, 2015), as well as to distinguish arm-reaching space from multisensory PPS (Zanini et al., 2021). Also, the experiment did not manipulate factors such as perspective (first- vs. third-person perspectives) and sense of agency (active vs. passive movements), both of which have been shown to differentially influence PPS and IPD (Candini et al., 2017; Iachini et al., 2014). Although BMI provides a valid proxy for body size, this construct may not fully capture different growth patterns across syndromes. However, using a virtual reachability task allowed us to compare PPS with IPD and to assess stimulus-specific modulations, in line with previous studies on action and social spaces (Nandrino et al., 2017; Cartaud et al., 2020; Iachini et al., 2014). Beyond addressing the methodological limitations discussed above, future research should also examine whether body-related spatial regulation in overgrowth syndromes changes across development, responds to psychosocial or rehabilitative intervention, and relates to real-world social participation and quality of life, extending the present cross-sectional, laboratory-based findings toward developmentally- and ecologically-informed models of multisensory body representation.

Despite these limitations, this study provides novel evidence that body-centered spatial representations differ in adolescents with overgrowth syndromes and that variability within this population is associated with cognitive functioning, body size, and social perception abilities. These findings contribute to a better understanding of how atypical bodily features and neurodevelopmental characteristics relate to multisensory spatial representation and social distance regulation during adolescence. Although clinical implications should be interpreted cautiously, the results highlight the importance of considering bodily and cognitive factors when addressing social challenges in individuals with overgrowth syndromes. Interventions targeting social participation in adolescents with overgrowth syndromes could benefit from explicitly addressing body-related spatial regulation, for instance through body-awareness or graded-exposure approaches, particularly for adolescents with BWSp and preserved cognitive functioning More broadly, by extending the study of body-related spatial representations to a novel population characterized by atypical physical growth and heterogeneous neurodevelopmental profiles, these findings contribute to the wider understanding of typical and atypical multisensory development, complementing evidence from sensory disability (Arioli et al., 2025) and ASD (Candini et al., 2020; Gessaroli et al., 2013).

## Data Availability

The analyzed dataset as well as the script and stimuli of the adopted task are freely available from OSF at the following link: https://osf.io/56kuc/overview?view_only=921cc633d286498dba42bc1229966337.
