# Supplementary material for: Peripersonal space and interpersonal distance as windows onto multisensory body representation in adolescents with overgrowth syndromes
Source: Front Hum Neurosci. 2026 Sep 8;20:1951893. doi: 10.3389/fnhum.2026.1951893 (PMC13593849; doi:10.3389/fnhum.2026.1951893)
Supplement: Supplementary file 1 [file Supplementary_file_1.docx]

**Supplementary analyses**

Within the clinical group, the preliminary ANOVA with sex as categorical factor revealed significant effects of task (*F_1,31_* = 36.30, *p* < .001, *η^2^_p_* = .54), stimulus (*F_2,62_* = 22.70, *p* < .001, *η^2^_p_* = .42), and their interaction (*F_2,62_* = 3.36, *p* = .041, *η^2^_p_* = .10), suggesting larger between-stimuli differences for IPD compared to PPS. However, in both tasks there were significant differences between cylinder, robot, and human stimuli (all *p* < .002). Crucially, neither the main effect of sex nor its interactions with task and stimulus were significant (all *p* > .113), ruling out potential confounding effects of sex on task performance.

Pearson’s correlation coefficients among the variables considered for the regression model are reported in Table S1.

**Table S1**. Correlations between variables considered for the regression model.

|  | **Age** | **AQ Social Skills** | **Total AQ** | **Theory of Mind** | **Affect Recognition** |
| --- | --- | --- | --- | --- | --- |
| **Age** | 1.000 | .132 | .142 | −.240 | −.264 |
| **AQ social skills** |  | 1.000 | .**782** | −.424 | −.319 |
| **Total AQ** |  |  | 1.000 | **−.709** | −.559 |
| **Theory of mind** |  |  |  | 1.000 | .**909** |
| **Affect recognition** |  |  |  |  | 1.000 |

As expected, strong associations were observed between the total AQ and the AQ Social Skills subscale, as well as between Theory of Mind and Affect Recognition subtests, indicating potential multicollinearity. To avoid redundancy among highly correlated measures (|*r*| > .60), only three predictors were retained for the multiple regression analysis: age, the AQ Social Skills subscale representing autism-like social difficulties, and Affect Recognition for social perception abilities.

All main analyses were repeated after excluding the three individuals with Malan syndrome. The ANCOVA comparing the clinical and control groups yielded results that closely mirrored those obtained in the full sample. The main effects of group (*F_1,60_* = 29, *p* < .001, *η^2^_p_* = .28), task (*F_1,60_* = 4.73, *p* = .034, *η^2^_p_* = .07) and stimulus (*F_2,120_* = 3.55, *p* = .032, *η^2^_p_* = .06) remained significant, as did the stimulus x group interaction (*F_2,120_* = 3.20, *p* = .044, *η^2^_p_* = .05). Neither the main effect of total AQ nor its interactions were significant (all *p* > .540).

When comparing participants with BWSp and those with Sotos syndrome, the 2 × 2 × 3 ANCOVA revealed significant main effects of task (*F_1,27_* = 12.61, *p* = .001, *η^2^_p_* = .32), as well as a significant task × group interaction (*F_1,31_* = 10.44, *p* = .003, *η^2^_p_* = .27). Post-hoc Duncan tests confirmed larger comfort than reachability distances across groups (all *p* < .006). No between-group differences emerged for IPD (*p* = .388; Sotos syndrome: 2.28 ± 0.11 vs. BWSp: 2.44 ± 0.15 m), while a trend towards greater PPS in Sotos syndrome was observed (*p* = .051; Sotos syndrome: 1.96 ± 0.11 vs. BWSp: 1.60 ± 0.14 m). Moreover, also the task × BMI interaction was significant (*F_1,27_* = 4.77, *p* = .038, *η^2^_p_* = .15), suggesting a differential impact of BMI on IPD and PPS. The follow-up Pearson correlations confirmed that BMI was positively associated with reaching distance (*r* = .41, *p* = .026), but not with comfort distance (*r* = -.01, *p* = .973).

After excluding the Malan subgroup, the ANCOVA examining intellectual level within the overgrowth syndromes group was also re-run. The pattern of results was consistent with that of the full clinical sample, with significant effects emerged for task (*F_1,27_* = 10, *p* = .004, *η^2^_p_* = .27) and task × group interaction (*F_1,27_* = 9.60, *p* = .005, *η^2^_p_* = 0.26), confirming a larger comfort distance in participants with average intellectual level compared to those with cognitive impairments. The main effect of the covariate BMI was not significant (*F_1,27_* = 2.46, *p* = .129, *η^2^_p_* = .08), although a trend towards significance was observed for the task × BMI interaction (*F_1,27_* = 4.05, *p* = .054, *η^2^_p_* = .13).

Consistent with the main analyses, no significant difference in BS emerged between adolescents with overgrowth syndromes and typically developing peers (*t*_61_ = −0.86, *p* = .392). Within the clinical sample, a significant difference in BS was observed between syndromic groups, with adolescents with BWSp showing larger BS values than those with Sotos syndrome (*t*_28_ = −2.76, *p* = .010). Similarly, adolescents with average intellectual functioning showed larger BS than those with cognitive impairments (*t*_28_ = −2.96, *p* = .006).

Lastly, the multiple regression model on BS was significant (Adjusted *R*^2^ = .26, *p* = .013), with affect recognition as significant predictor (*β* = .57, *p* = .002).

Overall, these supplementary analyses show that the main patterns observed in the full sample were preserved after exclusion of the Malan subgroup.
